# Zebrafish as a Model System for Investigating the Compensatory Regulation of Ionic Balance during Metabolic Acidosis

**DOI:** 10.3390/ijms19041087

**Published:** 2018-04-05

**Authors:** Lletta Lewis, Raymond W. M. Kwong

**Affiliations:** Department of Biology, York University, Toronto, ON M3J 1P3, Canada; lewisl@my.yorku.ca

**Keywords:** acidosis, zebrafish, ionic regulation, ionocytes, kidney

## Abstract

Zebrafish (*Danio rerio*) have become an important model for integrative physiological research. Zebrafish inhabit a hypo-osmotic environment; to maintain ionic and acid-base homeostasis, they must actively take up ions and secrete acid to the water. The gills in the adult and the skin at larval stage are the primary sites of ionic regulation in zebrafish. The uptake of ions in zebrafish is mediated by specific ion transporting cells termed ionocytes. Similarly, in mammals, ion reabsorption and acid excretion occur in specific cell types in the terminal region of the renal tubules (distal convoluted tubule and collecting duct). Previous studies have suggested that functional regulation of several ion transporters/channels in the zebrafish ionocytes resembles that in the mammalian renal cells. Additionally, several mechanisms involved in regulating the epithelial ion transport during metabolic acidosis are found to be similar between zebrafish and mammals. In this article, we systemically review the similarities and differences in ionic regulation between zebrafish and mammals during metabolic acidosis. We summarize the available information on the regulation of epithelial ion transporters during acidosis, with a focus on epithelial Na^+^, Cl^−^ and Ca^2+^ transporters in zebrafish ionocytes and mammalian renal cells. We also discuss the neuroendocrine responses to acid exposure, and their potential role in ionic compensation. Finally, we identify several knowledge gaps that would benefit from further study.

## 1. Introduction

Maintaining ionic and acid-base homeostasis is essential for all organisms. In recent years, the zebrafish (*Danio rerio*) has become an important model for understanding the mechanisms underlying the regulation of ionic and acid-base balance. Most fish species can only live in a narrow range of pH near neutrality, primarily owing to the disruption of ionic balance [1]. Zebrafish are highly tolerant to acid exposure (as low as pH 4.0), thus they can be useful for investigating the mechanisms underlying acid tolerance. Additionally, the zebrafish model has several advantages over other vertebrate models, including external and rapid development, short generation time, and applicability of many cellular and molecular physiological techniques in vivo (e.g., non-invasive measurements of ion and acid/base movement, whole-animal fluorescence imaging, transgenics, gene knockdown and knockout). 

Zebrafish inhabit a hypo-osmotic freshwater (FW) environment. To maintain ionic and acid-base homeostasis, they have to actively absorb ions and secrete acid to the environment. In adult zebrafish, the gills are the predominant site for regulating ion uptake through specific ion-transporting cells termed ionocytes. During larval stages before the gills are fully developed, regulation of ion transport is mediated by ionocytes found in the skin of the yolk sac. The transepithelial transport of major ions, such as Na^+^, Ca^2+^ and Cl^−^, occurs through three subtypes of ionocytes: H^+^-ATPase-rich cells (HRCs) for Na^+^ uptake, and for H^+^ and ammonia excretion; Na^+^-K^+^-ATPase-rich cells (NaRCs) for Ca^2+^ uptake; Na^+^-Cl^−^-cotransporter expressing cells (NCCCs) for both Na^+^ and Cl^−^ uptake. In the mammalian kidney, ion reabsorption and acid-base regulation are also mediated by specific cell types (Table 1). In mammals, most transepithelial H^+^ secretion occurs through the apical Na^+^/H^+^ exchanger 3 (NHE3) in the proximal tubule. The collecting duct also contributes significantly to H^+^ secretion through V type H^+^-ATPase expressed in Type A intercalated cells [2,3,4]. The expression (Table 1) and functional regulation (Table 2) of ion transporters/channels in the zebrafish ionocytes appear to be markedly similar to that in the mammalian renal cells. Therefore, the zebrafish gill or larval skin may serve as a useful in vivo model for investigating the mechanisms regulating ionic and acid-base balance during acid stress in vertebrates. 

The processes in the uptake and acid-base regulation in zebrafish have been comprehensively discussed in several previous reviews [1,5,6,7,8,9,10,11]. However, no article has systematically reviewed the similarities and differences in the regulation of epithelial ion transporters between zebrafish and mammals during metabolic acidosis. In this review, we summarize the available information on the regulation of ion transporters in zebrafish ionocytes and mammalian renal cells during acid stress. We discuss the neuroendocrine responses to acid exposure, and their potential role in ionic compensation. Finally, we identify areas that would benefit from future investigations with the zebrafish model.

## 2. Physiological Responses and Compensatory Regulation of Ion Transport during Acidosis

### 2.1. Overview of the Effects of Acid Exposure on Freshwater Fish

**Figure 1:**
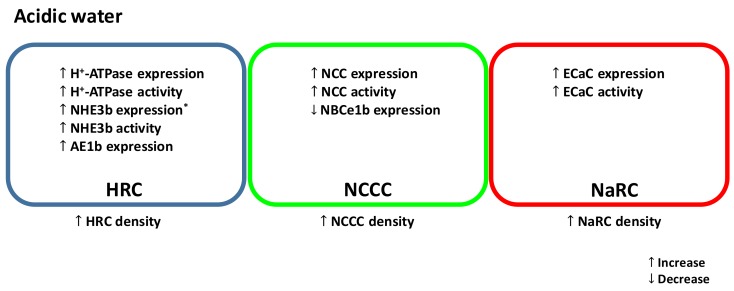
A simplified model illustrating the effects of acute and/or chronic acid exposure on zebrafish ionocytes. NHE, Na^+^/H^+^ exchanger; AE, anion exchanger; NCC, Na^+^-Cl^−^ cotransporter; NBCe, electrogenic Na^+^-HCO_3_^−^ cotransporter; ECaC, epithelial Ca^2+^ channel.HRC, H^+^-ATPase rich cell (blue); NCCC, NCC expressing cell (green); NaRC, Na^+^/K^+^-ATPase rich cell (red). * In acidic water, mRNA encoding NHE3b is increased in larvae but decreased in adult gills.

In most FW fish species studied, the primary effects of acid exposure are inhibition of active Na^+^ uptake coupled with stimulation of passive Na^+^ losses (for a review, see [1]). This disruption in Na^+^ regulation by acid exposure may result in a decrease in plasma Na^+^ level, ultimately leading to cardiovascular failure and mortality [51]. The reduced Na^+^ uptake by acid exposure is thought to occur through competitive inhibition with an elevated water H^+^ level [52]. Additionally, Na^+^ uptake in FW fish is predominately linked to H^+^ secretion through the actions of the Na^+^/H^+^ exchanger (NHE) and H^+^-ATPase at the apical membrane [53,54,55,56]. Therefore, the elevated level of H^+^ in the environment may reduce the gradient to drive Na^+^ influx. On the other hand, the increased Na^+^ efflux during acid exposure is believed to be associated with the disruption in the integrity of epithelial tight junction, thereby increasing the passive loss of Na^+^ through paracellular route [1]. Zebrafish, however, are able to increase active Na^+^ uptake and reduce paracellular Na^+^ loss following acid exposure. Several recent studies have demonstrated that zebrafish can also increase their capacity for Ca^2+^ and Cl^−^ uptake after acid exposure (discussed below). These compensatory responses are associated with an activation of a complex network of regulatory mechanisms, including modulation of the expression of ion transporters, stimulation of hormone production and thereby activation of its downstream signaling pathways, changes in the abundance of epithelial tight junction proteins, remodeling of ionocyte differentiation and proliferation, and post-translational regulation of ion transporters. A simplified model of the effects of acid exposure on zebrafish ionocytes is illustrated in Figure 1.

### 2.2. Functional Regulation of Ion Transporters and Their Involvement in Ionic Compensation during Acidosis

#### 2.2.1. Na^+^/H^+^ Exchanger (NHE)

In mammals, NHE3 (SLC9A3) is the major transporter regulating Na^+^ reabsorption in the proximal tubules of the kidney [57]. In the zebrafish gills/skin, NHE3 (i.e., NHE3b), which is specifically expressed in the apical membrane of HRCs, is also thought to be the predominant transporter for regulating Na^+^ uptake [17,42,58,59]. In the mammalian kidney, metabolic acidosis was reported to increase the abundance of NHE3 and its activity in the transport of Na^+^ [43]. In larval zebrafish, mRNA expression of *nhe3b* was found to increase following acid exposure [38]. However, its expression was decreased in the gills of adult zebrafish exposed to acidic water [17,48]. In acidic environments, elevated level of H^+^ in the water is expected to significantly impair Na^+^ uptake via NHE. Interestingly, NHE3b appears to play a role in increasing Na^+^ uptake after acid exposure. It has been suggested that NHE3b-mediated Na^+^ uptake in acidic water is overcome through its interaction with the ammonia transporter Rhcg1 [42]. Diffusion of NH_3_ through Rhcg1 creates a microenvironment where H^+^ level is lowered by its combination with NH_3_ to form NH_4_^+^. The resultant H^+^ gradient is believed to drive Na^+^ uptake via electroneutral NHE3b. However, recent findings have suggested that in acidic environments, the relative role of NHE3b is reduced and NCC may become the predominant pathway for the absorption of Na^+^ (discussed below). 

#### 2.2.2. Na^+^-Cl^−^ Cotransporter (NCC)

In the distal convoluted tubules of the mammalian kidney, NCC (SLC12A3) is responsible for fine-tuning the reabsorption of filtered Na^+^ (5%–7%) [60]. Several studies have investigated the effects of metabolic acidosis on the abundance and function of NCC. However, the findings are currently inconclusive. Following 7 days of metabolic acidosis, it was shown that the expression of NCC, as well as its activity in Na^+^ transport, were decreased in the rat kidney [61,62]. It was also demonstrated that the expression of NCC was increased significantly after 5 days of metabolic acidosis in both rat and mice kidney [47,63]. Clearly, further study is required to resolve this inconsistency regarding NCC expression during metabolic acidosis.

In zebrafish, NCC was found to be involved in the compensatory regulation of Na^+^ uptake following acid exposure [26,48]. In zebrafish, NCC (SLC12A10.2; an NCC-like member distinct from the mammalian orthologous of SLC12A3) is expressed in a specific subtype of ionocyte, the NCCCs [27]. Under normal condition, NHE3b plays a more critical role in Na^+^ regulation. However, recent studies have demonstrated that NCC could become important under physiological stressors, such as acidosis [26,48] or during periods of increased paracellular ion losses [64]. After acid exposure, the number of NCCCs in larval zebrafish skin as well as in the adult gills was markedly increased [48]. Whole body Na^+^ levels in NCC-deficient fish was also found to be lower than that in control fish following acid exposure [48]. Additionally, it was demonstrated that NCC was involved in the rapid regulation of both Na^+^ and Cl^−^ uptake after acute acidosis [26]. These responses appeared to compensate for the reduced Na^+^ and Cl^−^ uptake during acute acid exposure. Interestingly, this rapid compensation of Na^+^ and Cl^-^ uptake was found to be solely mediated by NCCCs, but not HRCs [26]. How NCC is acutely activated by acid exposure has remained unclear, but it has been proposed to occur via post-translational modification (e.g., phosphorylation) and/or by membrane trafficking [26]. A recent mammalian study has shown that post-translational activation of NCC is associated with the WNK4-SPAK signalling pathway [63]. Whether a similar mechanism also occurs in zebrafish NCC awaits further investigation. 

#### 2.2.3. Anion Exchanger (AE)

In mammals experiencing metabolic acidosis, bicarbonate excretion from the kidney is reduced to preserve base equivalents in the body. Such reduction is facilitated by two anion exchangers: pendrin and AE1. In the kidney, pendrin (SLC26A4; Cl^−^/HCO_3_^−^ exchanger) is expressed in the apical membrane of Type B intercalated cells. During metabolic acidosis, the expression of pendrin is significantly reduced [65,66]. Acid loading also appears to induce endocytosis of pendrin into the cytosol [46]. These responses subsequently decrease bicarbonate excretion into the lumen. SLC26A4 is also found to be expressed in zebrafish, and the knockdown of SLC26A4 significantly decreases Cl^-^ uptake after acclimation to low Cl^−^ water [67]. Reduced base excretion (i.e., titratable base) is also observed in SLC26A4-deficient zebrafish [67]. These findings indicate the physiological significance of SLC26A4 in Cl^−^ uptake and HCO_3_^−^ secretion in zebrafish. However, how acidosis affects the expression and function of SLC26A4 in zebrafish are yet to be explored.

AE1 (SLC4A), which is expressed in the basolateral membrane of Type A intercalated cells, exhibits opposite response during acidosis. Expression of AE1 is increased during metabolic acidosis, thereby enhancing bicarbonate absorption into the interstitium [22,68]. In zebrafish, AE1 (i.e., AE1b) is expressed in the basolateral membrane of HRCs [21]. It has been shown that acid exposure increases mRNA expression of *ae1b* in larval zebrafish [21]. Additionally, larval zebrafish experiencing AE1b knockdown have been found to exhibit a reduction in whole body Na^+^ level and H^+^ secretion [21]. These findings suggest that AE1b is involved in regulating Na^+^ balance and acid secretion in zebrafish. 

#### 2.2.4. Sodium-Bicarbonate Cotransporter (NBCe)

In mammals, the NBCe1 (“e” stands for electrogenic) plays a crucial role in maintaining normal blood pH by regulating bicarbonate absorption in the proximal tubules. The mammalian NBCe1 has multiple transcript variants, including NBCe1-A (kidney-specific), NBCe1-B (intestine/pancreas-specific) and NBCe1-C (brain-specific). Only NBCe1-A, which is expressed in the basolateral membrane of the proximal tubular cells, is thought to be involved in regulating bicarbonate absorption in the kidney [24,25]. It has been proposed that movement of HCO_3_^−^ via NBCe1-A is coupled to apical NHE3 and the activity of carbonic anhydrase (CA) [69]. Specifically, luminal bicarbonate is initially protonated by NHE3 and is converted to CO_2_ via a dehydration reaction that is accelerated by CA4. Dissolved CO_2_ permeates into the cell and undergoes a reverse hydration reaction catalyzed by CA2 which converts CO_3_^2−^ to HCO_3_^−^. Subsequently, the basolateral Na^+^/K^+^-ATPase generates the driving force for NBCe1-A mediated electrogenic Na^+^ and HCO_3_^-^ efflux. In rat, inducing metabolic acidosis by NH_4_Cl loading did not affect mRNA expression of NBCe1 in the kidney [70]. However, in isolated basolateral membrane vesicles from rabbit kidney, HCO_3_^−^-dependent Na^+^ uptake was increased during incubation in acidic media [71]. These results suggest that activation of the NBCe1-A by acid exposure is likely a post-transcriptional event. On the other hand, it has been documented that loss-of-function mutation in NBCe1-A results in proximal renal tubular acidosis [72,73,74]. NBCe1 null mice (NBCe1^−/−^) have also been found to exhibit severe metabolic acidosis and death before weaning [75].

In zebrafish, the NBCe1 protein sequence is more similar to the mammalian NBCe1-B (78%) than NBCe1-A (73%) [21,23]. Zebrafish NBCe1-B is expressed in the pronephros, eyes, brain, and ionocytes (i.e., NCCCs) in larvae, as well as the gills in adults [21,23]. Using a *Xenopus* oocyte expression system, it was demonstrated that zebrafish NBCe1-B exhibited electrogenic Na^+^ and HCO_3_^−^ cotransport activity [23]. As discussed previously, the transport function of NBCe1 is believed to be coupled with the activity of NHE3 and CA2. In zebrafish, these proteins (i.e., NHE3b, CA2 like-a) are expressed specifically in the HRCs [18,59,76]. Interestingly, NBCe1-B was shown to be expressed in NCCCs [21]. In the gills of zebrafish, mRNA level of NBCe1-B was also found to decrease following acid exposure [21]. These results suggest that NBCe1-B is unlikely involved in bicarbonate absorption in zebrafish. The functional role of NBCe1-B in the basolateral membrane of NCCCs remains to be explored.

#### 2.2.5. Epithelial Ca^2+^ Channel (ECaC)

In mammals, transepithelial transport of Ca^2+^ is mediated by the transient receptor potential channels (TRPV): TRPV5 (also called ECaC) and TRPV6. TRPV5 contributes to Ca^2+^ transport in the kidney, while TRPV6 is more ubiquitously expressed [77]. In the mammalian kidney, the majority of Ca^2+^ reabsorption occurs in the proximal tubules (~60–70%) and the thick ascending limb of Henle (~10%) through the paracellular route [78]. This passive movement of Ca^2+^ is driven by local Na^+^ reabsorption, and thus Ca^2+^ reabsorption in these regions is not directly regulated. Although only 5–10% of total Ca^2+^ reabsorption occurs in the distal convoluted tubule and the collecting duct, these are the major sites for active regulation of Ca^2+^ transport by TRPV5 [78,79].

The uptake of Ca^2+^ by ECaC in the mammalian kidney is highly influenced by luminal pH. For example, it was shown that a decrease in extracellular pH significantly inhibited Ca^2+^ uptake in primary cultures from rabbit cortical collecting duct [80]. Additionally, reduced pH was also demonstrated to inhibit Ca^2+^ uptake in a human embryonic kidney 293 cell line (HEK 293) expressing ECaC [81]. 

Unlike mammals, zebrafish only have one TRPV5/6 orthologue (ECaC) [31]. In zebrafish, ECaC is expressed in a subset of NaRCs, which plays an essential role in Ca^2+^ absorption [31,82]. Previous studies have shown that larval zebrafish exposed to acidic water exhibit a significant reduction in whole body Ca^2+^ level [39,49]. Such reduction is likely due to a concurrent decrease in Ca^2+^ uptake and an increase in Ca^2+^ efflux [49]. Unlike Na^+^ uptake, which is stimulated in zebrafish following acid acclimation, Ca^2+^ uptake remains significantly inhibited in acidic water [49]. Interestingly, when the acid-exposed fish are returned to control pH water, the capacity for Ca^2+^ uptake is substantially increased. The increased Ca^2+^ uptake appears to be associated with an increased mRNA expression of *ecac* and density of *ecac*-expressing NaRCs [49]. This finding appears to be different from a previous study with mice where acid-loading (NH_4_Cl) resulted in a reduction in *ecac* expression in the kidney [50]. The mechanisms underlying the increased *ecac*-expressing NaRCs by acid exposure in zebrafish remain unknown. Nevertheless, acid exposure has been shown to increase whole body cortisol level in zebrafish [49,83], and that cortisol is involved in promoting the proliferation of NaRCs [84,85]. Therefore, it is possible that the increased *ecac*-expressing NaRCs is due to the increased cortisol level during acid exposure. On the other hand, it has been reported that exposure to acidic water increases whole body 3′,5′-cyclic adenosine monophosphate (cAMP) levels in zebrafish [86], and that treatment with cAMP-elevating agents (i.e., 8-Br-cAMP, forskolin) significantly stimulates Ca^2+^ influx [87]. Future experiments should address whether cAMP plays a role in stimulating Ca^2+^ uptake following acid exposure. It is also noteworthy that zebrafish exposed to acidic water for 4 days exhibited over a 5-fold reduction in whole body Ca^2+^ level [39,49]. The increased Ca^2+^ uptake by acid exposure was only observed when the fish were returned back to normal pH water [49]. How zebrafish compensate for the loss of Ca^2+^ (e.g., regulation of paracellular permeability to Ca^2+^) during long-term acid exposure requires further investigation.

#### 2.2.6. Effects of Acidosis on the Regulation of Other Epithelial Ion Transporters

In mammals, several other epithelial ion transporters are known to be involved in ionic compensation during metabolic acidosis, including Na^+^/phosphate cotransporter (NaPi; Pi stands for inorganic phosphate), Cl^−^/HCO_3_^−^ exchanger, and H^+^-K^+^-ATPase (HKA). However, whether these transporters are involved in ion regulation in zebrafish exposed to acidic water are virtually unknown. In mammals, NaPi has three different protein families, NaPi-I, NaPi-II and NaPi-III, with NaPi-II thought to be the major player in Pi regulation in the kidney [88]. In the mammalian kidney, reabsorption of Pi occurs predominantly in the proximal tubule [89]. In rats experiencing 10 days of metabolic acidosis, renal mRNA and protein expression of NaPi (NaPi-II) were significantly decreased [90]. The transport activity (e.g., Jmax) of NaPi was also found to decrease [90]. In another study with the mouse kidney, however, it was demonstrated that NaPi (NaPi-IIa and NaPi-IIc) protein abundance was increased after 2 days or 10 days of acidosis [91]. The activity of NaPi was found to increase after 2 days of acidosis, but then returned to normal level after 7 days [91]. These findings suggest possible species-specific differences in the regulation of NaPi during acid exposure. In zebrafish, two paralogues of NaPi-IIb, NaPi-IIb1 and NaPi-IIb2, were found to express in the apical membrane of renal tubular cells [92]. NaPi-IIb1 and NaPi-IIb2 appeared to exhibit a significant difference in their functional properties. For example, the transport activity of zebrafish NaPi-IIb2 was shown to have a much higher affinity to Pi than NaPi-IIb1 when expressed in *Xenopus* oocytes [92]. NaPi-IIb2 was also found to be insensitive to extracellular pH as opposed to NaPi-IIb1 [92]. To date, no study has yet identified the expression of NaPi in zebrafish gill/ionocytes. [93,94]

In mammals, excretion of inorganic sulfate (Si) is primarily regulated by the Na-Si cotransporter in the proximal tubule [95]. During metabolic acidosis, reabsorption of Si is decreased [96], which appears to be associated with decreased NaSi-1 mRNA and protein abundance, as well as reduced Na^+^-Si cotransport activity [96]. In zebrafish, NaSi1 mRNA is expressed in the kidney [97,98]. Functional characterization of the zebrafish NaSi1 in *Xenopus* oocytes suggested that the zebrafish transporter closely resembles the mammalian counterpart in terms of both affinity and transport capacity [97]. However, NaSi1 was not found to be expressed in the zebrafish gill [97]. 

In the mammalian kidney, K^+^ reabsorption and acid excretion can be mediated by HKA, which is predominantly expressed in the apical membrane of intercalated cells (both type-A and type-B) and principal cells [99]. Mice experiencing metabolic acidosis exhibited an increased mRNA expression of HKA (α-subunits) [100]. The activity of HKA was also found to increase during metabolic acidosis [101,102]. The protein sequence of the mammalian H^+^-K^+^-ATPase α-subunit is most similar to the zebrafish Na^+^/K^+^-ATPase α-subunit isoforms 1, 2, 3, 5 and 8 (~63%–64%). Currently, however, no physiological evidence has demonstrated that these zebrafish Na^+^/K^+^-ATPases can function as HKA.

## 3. Neuroendocrine Responses Following Acid Exposure

In zebrafish, several neuroendocrine factors are found to be involved in ionic and acid-base regulation during acid exposure, including cortisol [38,83], endothelin 1 [103], angiotensin II [104], stanniocalcin [49,105], catecholamines [106] and oestrogen-related receptor [107]. In other FW fish species, mRNA expression and/or plasma levels of thyroid hormone [108], prolactin [109], growth hormone [110] and somatolactin [110,111] are found to be modulated by acid exposure. Whether these hormones are affected by acid exposure in zebrafish remain unexplored.

### 3.1. Cortisol

In mammals, the corticosteroid is a class of steroid hormones that include cortisol (glucocorticoid) and aldosterone (mineralocorticoid). Both of these hormones are synthesized in the adrenal cortex of mammals, with the aldosterone thought to be the primary factor in regulating ion reabsorption [112]. In mammals, activation of the mineralocorticoid receptor (MR) stimulates Na^+^ reabsorption and H^+^ excretion in the kidney [113]. Teleosts lack aldosterone synthase to produce aldosterone [114]. However, several in vitro studies have demonstrated that cortisol is able to activate both glucocorticoid receptor (GR) and MR [114,115,116]. 

In zebrafish larvae, GR was found to be expressed in all three ionocytes discussed above, including NaRCs [85], HRCs [83,117] and NCCCs [118]. Treatment with cortisol was shown to increase mRNA expression of genes that are involved in ion uptake or H^+^ secretion, including *ecac*, *ncc*, *nhe3b*, *atp6v1a* (encodes for H^+^-ATPase) and *ae1b* [38,117]. On the other hand, mRNA level of 11β-hydroxylase (*cyp11b*; encodes for a cortisol-synthesis enzyme) was found to increase following acid exposure [38]. Consistently, whole body level of cortisol was markedly increased in larval zebrafish exposed to acidic water [49,83]. The increased whole body cortisol level by acid exposure appears to be important in increasing acid secretion and active Na^+^ uptake in the HRCs [38,83,119]. During acid exposure, cortisol was also shown to be involved in reducing diffusive Na^+^ losses through paracellular pathways, likely by increasing the abundance of epithelial tight junctions [119]. Interestingly, the cortisol-induced effects on Na^+^ regulation and H^+^ secretion in zebrafish appear to be mediated by GR only. 

### 3.2. Endothelin

In mammals, endothelin is involved in increasing acid excretion in the kidney during metabolic acid stress [120]. During acid loading, expression of endothelin is increased in the kidney [121], which subsequently stimulates acid secretion by enhancing the activity of NHE3 [122,123] and H^+^-ATPase [124]. A recent study with zebrafish has also suggested that endothelin plays a critical role in regulating acid secretion [103]. The mRNA expression levels of endothelin-1 (*edn1*) and its receptors (*ednraa*) were found to increase in zebrafish exposed to acidic water. EDN1 was also shown to promote acid secretion, primarily by post-translational activation of the H^+^-ATPase in the HRCs of zebrafish [103]. These findings clearly demonstrate that endothelin is involved in regulating acid excretion in zebrafish.

### 3.3. Oestrogen-Related Receptor

The oestrogen-related receptor (ERR) belongs to orphan nuclear receptors which control the transcription of several genes essential in energy metabolism in mammals [125]. In zebrafish, it was recently demonstrated that ERRa is involved in promoting acid secretion in zebrafish, partially by increasing the density of HRCs [107]. *erra* mRNA was found to be expressed in HRCs, and that acid exposure increased its abundance [107]. ERRa was also demonstrated to positively regulate the mRNA expressions of *nhe3b*, *atp6v1a* (encodes for H^+^-ATPase) and *ae1b* [107]. This is a novel finding on the role of ERRa in acid-base regulation in vertebrates.

### 3.4. Catecholamines

In mammals, it is well documented that catecholamines are involved in regulating Na^+^ reabsorption in the kidney [126]. For example, treatment with catecholamines was found to stimulate Na^+^/K^+^-ATPase activity in the basolateral membrane of proximal tubules isolated from rats [127,128]. The activity and abundance of NHE3 in the proximal tubule of rats were also shown to increase following stimulation of the α-adrenergic receptor [129,130,131]. It was also demonstrated that activation of the β adrenergic receptor increased NHE3 activity by promoting the association of the NHE regulatory factor (NHERF) with NHE3 [132]. At present, the involvement of the adrenergic system in the kidney during metabolic acidosis is not fully understood.

In larval zebrafish, both HRCs and NaRCs are innervated, and β receptors appear to be present in both cell types [7,106,133]. Whether NCCCs are innervated is currently unknown. It has been demonstrated that knockdown of specific β adrenergic receptor reduces the magnitude of increased Na^+^ uptake in larval zebrafish exposed to acidic water [106]. The underlying mechanism of how β adrenergic receptor elicits its effect on Na^+^ transporters remains unclear. The downstream signalling cascade of β adrenergic receptor is mediated by cAMP, and it has been shown that treatment with cAMP-elevating agents increases Na^+^ uptake in larval zebrafish [86]. Whole body cAMP level has also been found to increase in larval zebrafish acutely exposed to acidic water [86]. These findings suggest that activation of the β adrenergic system during acid exposure increases whole body cAMP level, which subsequently increases Na^+^ uptake. Using a morpholino knockdown approach, it has also been demonstrated that cAMP-stimulated Na^+^ uptake is likely mediated by both NHE3b and NCC [86]. 

### 3.5. Angiotensin II

In mammals, the renin-angiotensin system (RAS) plays a critical role in regulating ion and fluid reabsorption in the kidney [134]. Angiotensinogen, which is primarily released from the liver, is first cleaved to angiotensin-I (ANG-I) by the enzyme renin secreted in the kidney. ANG-I is then converted to the biologically active form ANG-II by angiotensin-converting enzyme (ACE). In the mammalian kidney, ANG-II is found to stimulate NHE3, H^+^-ATPase and NCC [135,136,137,138,139,140]. A previous study with larval zebrafish has demonstrated that whole body ANG-II level is elevated following acid exposure [104]. It has also been shown that translational gene knockdown of renin significantly attenuated the increased Na^+^ uptake by acute acid exposure [104]. These findings suggest that the RAS is involved in promoting Na^+^ uptake under acidic environment. On the other hand, treatment with exogenous ANG-II was found to increase the mRNA expression of *ncc*, but not *nhe3b* or *atp6v1a* (encodes for H^+^-ATPase) [104]. Interestingly, NCC appears to play a critical role in the rapid compensatory regulation of Na^+^ uptake following acute acid exposure [26]. In mammals, ANG-II is proposed to activate NCC through post-translational regulation [141]. Whether similar mechanism occurs in zebrafish NCC has yet to be explored. The expression of ANG-II receptors in ionocytes of zebrafish also awaits further study.

### 3.6. Stanniocalcin

In mammals, the principal cells in the collecting duct are one of the primary sources of stanniocalcin, which acts as a hypocalcemic factor to decrease Ca^2+^ uptake in the intestine and kidney [142,143]. In zebrafish, stanniocalcin (i.e., STC1) is known to negatively regulate *ecac* expression and thereby reducing Ca^2+^ uptake [144]. Recently, this hormone has also been proposed to regulate acid excretion and Ca^2+^ transport following acid exposure [49,105]. Exposure to acidic water appears to specifically reduce mRNA expression of *stc1*, but not other Ca^2+^-regulatory hormones (e.g., parathyroid hormone, calcitonin) [49,105]. The reduced expression of *stc1* may potentially promote Ca^2+^ uptake by increasing *ecac* expression and density of *ecac*-expressing NaRCs during acid exposure [49,105]. Additionally, acid excretion is significantly reduced in zebrafish overexpressing STC1, possibly owing to its inhibitory effects on HRCs differentiation from epidermal stem cells [105]. These novel findings from the zebrafish model provide new understanding of the role of STC1 during metabolic acidosis.

## 4. Conclusions and Perspectives

The zebrafish has emerged as a useful in vivo model to investigate the cellular and molecular mechanisms underlying the regulation of ionic and acid-base homeostasis in vertebrates. Previous research efforts with the gill/skin of zebrafish have greatly improved our fundamental understanding of the compensatory mechanisms, ion transport function, and neuroendocrine responses during metabolic acidosis. However, it is also important to recognize the differences in the expression pattern and functional regulation of some of the ion transporters between zebrafish and higher vertebrates. For examples, (i) H^+^-ATPase is expressed in both the apical and basolateral membranes of mammalian intercalated cells, whereas it is expressed only in the apical membrane of zebrafish HRCs; (ii) NaPi and NaSi are absent in the zebrafish gill/ionocytes; (iii) compared to zebrafish, mammals do not appear to compensate for the increased Ca^2+^ loss during acid loading. These differences may ultimately contribute to different strategies employed by zebrafish and mammals in regulating systemic acid-base and ion homeostasis during metabolic acidosis.

Currently, studies on the ionic and acid-base regulation in zebrafish are mostly limited to early developmental stages. Ion regulation occurs in the skin of the yolk sac in larvae, as opposed to the gills, intestine, and kidney in adults. Therefore, future studies should also address the regulation of ionic and acid-base balance in adult zebrafish. Additionally, a number of areas still remain largely unknown, and should be addressed in future investigations: (i) identification of the localization of several hormone receptors (e.g., receptors for STC1 and ANG-II) in zebrafish ionocytes; (ii) mechanism of environmental and intracellular acid sensing; (iii) responses of other neuroendocrine factors that are thought to be important in ionic and acid-base regulation in FW fish (e.g., thyroid hormone, prolactin, growth hormone, and somatolactin); (iv) post-translational regulation of ion transporters/channels; (v) potential crosstalk among neuroendocrine factors; and (vi) role of transcription factors and hormones in ionocyte differentiation/proliferation during acidosis. Furthermore, previous research on the effects of acidosis in zebrafish is limited to Na^+^ and Ca^2+^, and to a lesser extent Cl^−^. Future studies should also address the regulation of other major ions (e.g., Mg^2+^ and K^+^) during acid exposure.

## Figures and Tables

**Table 1:** Distribution of major epithelial ion transporters in zebrafish ionocytes and mammalian renal cells.

Transporters	Gene Name and Cellular Localization of the Protein		Protein Identity between Zebrafish and Humans
	Zebrafish	Mammals	
H^+^-ATPase	ATP6V1AA. HRCs (apical) [12,13]	ATP6V1A. Proximal tubular cells (apical), Type A (apical) and type B (basolateral) intercalated cells [14,15,16]	93%
NHE3	NHE3b; SLC9A3.2. HRCs (apical) [17,18]	NHE3; SLC9A3. Proximal tubular cells (apical) [19,20]	47%
AE1	AE1b; SLC4A1B. HRCs (basolateral) [21]	AE1; SLC4A1. Type A intercalated cells (basolateral) [22]	53%
NBCe1	NBCe1b; SLC4A4B. NCCCs (basolateral) [21,23]	NBCe1-A ^‡^; SLC4A4. Proximal tubular cells (basolateral) [24,25]	78%
NCC	NCC like 2; SLC12A10.2. NCCCs (apical) [26,27]	NCC; SLC12A3. Distal convoluted tubular cells (apical) [28,29,30]	53%
ECaC	ECaC; TRPV5. NaRCs (apical) [31,32]	ECaC; TRPV5. Distal convoluted tubular cells and principal cells (apical) [33,34,35]	48%
ENaC	N/A *	ENaC. Principal cells (apical) [36,37]	N/A *

NHE, Na^+^/H^+^ exchanger; AE, anion exchanger; NBCe, electrogenic Na^+^-HCO_3_^−^ cotransporter; NCC, Na^+^-Cl^−^ cotransporter; ECaC, epithelial Ca^2+^ channel; ENaC, epithelial Na^+^ channel. HRCs, H^+^-ATPase-rich cells; NCCCs, Na^+^-Cl^−^ cotransporter expressing cells; NaRCs, Na^+^/K^+^-ATPase-rich cells. TRPV, transient receptor potential cation channel. * The gene encoding for ENaC has not been identified in teleosts. ^‡^ NBCe1-A is a kidney-specific splice variant of NBCe1.

**Table 2:** The in vivo effects of acid exposure on the regulation of major epithelial ion transporters in zebrafish ionocytes and mammalian renal cells.

Ion Transporters	Expression Levels/Activity	
	Zebrafish *	Mammals
H^+^-ATPase	Chronic: ↑ H^+^-ATPase mRNA expression ↑ H^+^-ATPase activity ↑ HRCs density [12,38,39]	Acute: ↑ H^+^-ATPase mRNA (rabbit) [40]
		Chronic: ↑ H^+^-ATPase protein abundance (rabbit) [41]
NHE3	Chronic: ↑ *nhe3b* mRNA expression [38] ↑ NHE3b activity [42]	Chronic: ↑ NHE3 protein abundance (rat) [43,44] ↑ NHE activity (rat) [45]
AE1	Chronic: ↑ *ae1b* mRNA expression [38]	Chronic ↑ AE1-expressing cells ↑ AE1 protein abundance (rabbit) [46]
NCC	Acute: ↔ *ncc* mRNA expression ↔ NCCCs density ↑ NCC activity [26]	Acute: ↔ NCC protein abundance (rat) [47]
	Chronic: ↑ *ncc* mRNA expression ↑ NCCCs density [48]	Chronic: ↑ NCC protein abundance (rat) [47]
ECaC	Chronic: ↑ *ecac*-expressing cells ↑ ECaC activity [49]	Chronic: ↓ *ecac* (TRPV5) mRNA expression ↓ ECaC protein abundance (mice) [50]

↑ Increase, ↓ decrease, ↔ no change. * Regulation of ion transporters at larval stages (0 to 4 days post-fertilization). Acute: ≤24 h of exposure, chronic: >1 day of exposure.
